# Comparison of landiolol and amiodarone for the treatment of new-onset atrial fibrillation after cardiac surgery (FAAC) trial: study protocol for a randomized controlled trial

**DOI:** 10.1186/s13063-023-07353-6

**Published:** 2023-05-25

**Authors:** Edouard Caspersen, Pierre-Grégoire Guinot, Bertrand Rozec, Jean-Ferréol Oilleau, Jean-Luc Fellahi, Philippe Gaudard, Emmanuel Lorne, Yazine Mahjoub, Emmanuel Besnier, Mouhamed Djahoum Moussa, Nicolas Mongardon, Jean-Luc Hanouz, Anaïs R. Briant, Laure Peyro Saint Paul, Clémence Tomadesso, Jean-Jacques Parienti, Richard Descamps, Alina Denisenko, Marc-Olivier Fischer, for the ARCOTHOVA Group

**Affiliations:** 1 Department of Anaesthesiology-Resuscitation and Perioperative Medicine, Normandy University, UNICAEN, Caen University Hospital, Normandy, Caen, France; 2 Department of Anaesthesiology and Intensive Care, Dijon University Hospital, Dijon, France; 3 University of Bourgogne and Franche-Comté, LNC UMR1231, Dijon, France; 4 INSERM, LNC UMR1231 Dijon, France; 5 LipSTIC LabEx, FCS Bourgogne-Franche Comté, Dijon, France; 6 Service d’Anesthésie-Réanimation, Hôpital Laennec, CHU Nantes, Nantes, France; 7 Institut du Thorax, Université de Nantes, CHU Nantes, CNRS, INSERM, Nantes, France; 8 Department of Anesthesiology and Surgical Intensive Care Unit, Brest University Hospital, 29200 Brest, France; 9 Service d’Anesthésie-Réanimation, Hôpital Universitaire Louis Pradel, Hospices Civils de Lyon, Lyon, France; 10 Faculté de Médecine Lyon Est, Université Claude-Bernard, Lyon 1, Lyon, France; 11 Department of Anesthesiology and Critical Care Medicine Arnaud de Villeneuve, CHU Montpellier, University of Montpellier, Montpellier, France; 12 Department of Anaesthesia and Critical Care Medicine, Clinique du Millénaire, Cedex 2, 34960 Montpellier, France; 13 Anesthesia and Critical Care Department, Amiens Hospital University, Amiens, France; 14 Department of Anesthesiology and Critical Care, Rouen University Hospital, Rouen, France; 15 Univ. Lille, Inserm, CHU Lille, Institut Pasteur de Lille, U1011- EGID, F-59000 Lille, France; 16 Université Paris-Est Creteil Val de Marne, 27010 Creteil, France; 17 Department of Biostatistics, Normandy University, UNICAEN, Caen University Hospital, Normandy, Caen, France; 18 Department of Clinical Research and Innovation, Normandy University, UNICAEN, Caen University Hospital, Normandy, Caen, France; 19 Institut Aquitain du Coeur, Clinique Saint Augustin, Elsan, 114 Avenue d’Arès, 33074 Bordeaux Cedex, France

**Keywords:** Anti-arrhythmic drugs, Anti-coagulation, Amiodarone, Atrial fibrillation, Beta-blockers, Cardiac surgery

## Abstract

**Background:**

Postoperative atrial fibrillation (PoAF) after cardiac surgery has a high incidence of 30%, but its management is controversial. Two strategies are recommended without evidence of a superiority of one against the other: rate control with beta-blocker or rhythm control with amiodarone. Landiolol is a new-generation beta-blocker with fast onset and short half-life. One retrospective, single-center study compared landiolol to amiodarone for PoAF after cardiac surgery with a better hemodynamic stability and a higher rate of reduction to sinus rhythm with landiolol, justifying the need for a multicenter randomized controlled trial. Our aim is to compare landiolol to amiodarone in the setting of PoAF after cardiac surgery with the hypothesis of a higher rate of reduction to sinus rhythm with landiolol during the 48 h after the first episode of POAF.

**Methods:**

The FAAC trial is a multicenter single-blind two parallel-arm randomized study, which planned to include 350 patients with a first episode of PoAF following cardiac surgery. The duration of the study is 2 years. The patients are randomized in two arms: a landiolol group and an amiodarone group.

Randomization (Ennov Clinical®) is performed by the anesthesiologist in charge of the patient if PoAF is persistent for at least 30 min after correction of hypovolemia, dyskalemia, and absence of pericardial effusion on a transthoracic echocardiography done at bedside.

Our hypothesis is an increase of the percentage of patients in sinus rhythm from 70 to 85% with landiolol in less than 48 h after onset of PoAF (alpha risk = 5%, power = 90%, bilateral test).

**Discussion:**

The FAAC trial was approved by the Ethics Committee of EST III with approval number 19.05.08. The FAAC trial is the first randomized controlled trial comparing landiolol to amiodarone for PoAF after cardiac surgery. In case of higher rate of reduction with landiolol, this beta-blocker could be the drug of choice used in this context as to reduce the need for anticoagulant therapy and reduce the risk of complications of anticoagulant therapy for patients with a first episode of postoperative atrial fibrillation after cardiac surgery.

**Trial registration:**

ClinicalTrials.gov NCT04223739. Registered on January 10, 2020.

**Supplementary Information:**

The online version contains supplementary material available at 10.1186/s13063-023-07353-6.

## Administrative information

**Table:** 

Title {1}	Comparison of landiolol and amiodarone for the treatment of new-onset atrial fibrillation after cardiac surgery (FAAC) trial: study protocol for a randomized controlled trial
Trial registration {2a and 2b}	ClinicalTrials.gov Identifier: NCT04223739 (Registered January 10, 2020) Registration name: Comparison of two strategies for the management of atrial fibrillation after cardiac surgery
Protocol version {3}	Version 5 from 28/09/2021
Funding {4}	Health French ministry (Direction Générale de l’Offre de Soins DGOS) and AOP ORPHAN PHARMACEUTICALS. Funders had no role in study design; in the collection, analysis and interpretation of data; in the writing of the report; or in the decision to submit the report for publication
Author details {5a}	*Edouard Caspersen*^*1*^*, Pierre-Grégoire Guinot*^*2*^*, Bertrand Rozec*^*3*^*, Jean-Ferréol Oilleau*^*4*^*, Jean-Luc Fellahi*^*5*^*, Philippe Gaudard*^*6*^*, Emmanuel Lorne*^*7*^*, Yazine Mahjoub*^*8*^*, Emmanuel Besnier*^*9*^*, Mouhamed Djahoum Moussa*^*10*^*, Nicolas Mongardon*^*11*^*, Jean-Luc Hanouz*^*1*^*, Anaïs R. Briant*^*12*^*, Clémence Tomadesso*^*13*^*, Jean-Jacques Parienti*^*12*^*, Richard Descamps*^*1*^*, Alina Denisenko*^*1*^*, Marc-Olivier Fischer*^*14*^ ^*1*^ Department of Anaesthesiology-Resuscitation and Perioperative Medicine, Normandy University, UNICAEN, Caen University Hospital, Normandy, Caen, France 2 Department of Anaesthesiology and Intensive Care, Dijon University Hospital, Dijon, France; University of Bourgogne and Franche-Comté, LNC UMR1231, Dijon, France; INSERM, LNC UMR1231, Dijon, France; FCS Bourgogne-Franche Comté, LipSTIC LabEx, Dijon, France 3 Service d'Anesthésie-Réanimation, Hôpital Laennec, CHU Nantes, Nantes, France; Université de Nantes, CHU Nantes, CNRS, INSERM, Institut du Thorax, Nantes, France 4 Department of Anesthesiology and Surgical intensive care unit, Brest University Hospital, 29,200 Brest, France 5 Service d'Anesthésie-Réanimation, Hôpital Universitaire Louis Pradel, Hospices Civils de Lyon, Lyon, France; Faculté de Médecine Lyon Est, Université Claude-Bernard Lyon 1, Lyon, France 6 Department of Anesthesiology and Critical Care Medicine Arnaud de Villeneuve, CHU Montpellier, University of Montpellier, Montpellier, France 7 Department of Anaesthesia and Critical Care Medicine, Clinique du Millénaire, 34,960 Montpellier Cedex 2, France 8 Anesthesia and Critical Care Department, Amiens Hospital University, Amiens, France 9 Department of Anesthesiology and Critical Care, Rouen University Hospital, Rouen, France 10 Univ. Lille, Inserm, CHU Lille, Institut Pasteur de Lille, U1011- EGID, F-59000, Lille, France 11 Université Paris-Est Creteil Val de Marne, 27,010, Creteil, France 12 Department of Biostatistics, Normandy University, UNICAEN, Caen University Hospital, Normandy, Caen, France 13 Department of Clinical Research and Innovation, Normandy University, UNICAEN, Caen University Hospital, Normandy, Caen, France 14 Institut Aquitain du Coeur, Clinique Saint Augustin, Elsan, 114 avenue d’Arès, 33 074 Bordeaux cedex
Name and contact information for the trial sponsor {5b}	University Hospital of Caen, Research and Innovation Department, Avenue de la Côte de Nacre, 14,033 CAEN CEDEX Phone: + 332 31 06 57 81 Email: tomadesso-c@chu-caen.fr
Role of sponsor {5c}	The sponsor is the University Hospital of Caen, an academic hospital, responsible for protocol decisions. Their roles are firstly to insure patient’s right protection and data validity via quality control of the trial. In addition to management, analysis and interpretation of data, assistance in writing of the report and support in the publication. Data collected are sponsor property and every project of publication have to be validated by sponsor before submission and include sponsor in authors

## Introduction

### Background and rationale {6a}

Postoperative atrial fibrillation (PoAF), defined as new-onset atrial fibrillation in the immediate postoperative period, is a clinically relevant problem occurring in 20 to 50% of patients after cardiac surgery [1]. Intra- and postoperative changes affecting atrial fibrillation triggers and pre-existing atrial substrate may increase atrial vulnerability to arrhythmia. PoAF has been associated with hemodynamic instability, prolonged hospital stays, infections, renal complications, bleeding, increased in-hospital death, and greater healthcare costs, and it has been shown to be a risk factor for stroke [1]. However, its management is controversial. Two therapeutic strategies are recommended without evidence of a superiority of one against the other [1–3]: the rate control with negative chronotropic agents as beta-blockers or rhythm control with amiodarone as an anti-arrhythmic agent. A study by Gillinov et al. showed no difference in complications and duration of hospital stay between these two strategies [4]. However, this study suffered from some limitations as the modality of beta-blocker use (oral route, no predetermined administration plan, goal heart rate of 100/min which is still high concerning myocardial energy balance) [5]. Landiolol is a new generation of intra-venous beta-blocker with fast onset (1 min) and short half-life (4 min). Landiolol has been used for over 20 years in Japan [6], and its use has recently been approved in Europa for perioperative supraventricular arrhythmia. Due to its S-Enantiomer conformation, landiolol has less of a negative inotropic effect than esmolol (only other available intravenous beta-blocker) [7]. One study compared landiolol to amiodarone for PoAF after cardiac surgery with a better hemodynamic stability (less bradycardia and hypotension) and a higher rate of reduction to sinus rhythm with landiolol [8]. However, this was a retrospective, single-center study including few patients [8].

In this protocol, we describe the design of a multicenter randomized controlled trial to compare landiolol to amiodarone for the treatment of new-onset PoAF after cardiac surgery.

### Objectives {7}

The primary objective is the number of patients in sinus rhythm 48 h after the first episode of POAF after cardiac surgery. The secondary objectives are the hemodynamic tolerance, the ICU and hospital length of stay, and the recurrence of POAF and/or thromboembolic complications and/or hemorrhagic complications and/or number of patients with an adverse event due to landiolol or amiodarone, within 2 months and 1 year after surgery. Table 1 summarizes the objectives and time points.

**Table 1:** Objectives

	Outcomes	Measurements	Time points
Primary	Proportion of patient in sinus rhythm at 48 h	Number (percentage) of patients	48 h
Secondary	Hemodynamic tolerance of treatment	Incidence of MAP < 60 mmHg and/or bradycardia as heart rate < 40/min	ICU LOS
	ICU LOS	Total days	ICU LOS
	Hospital LOS	Total days	Hospital LOS
	Recurrence of PoAF	EKG	2 months and 1 year
	Thromboembolic complications	Stroke or embolic ischemia	2 months and 1 year
	Hemorrhagic complications	Active bleeding uncontrollable, with hemodynamic instability, with an urgency treatment, or in a location with functional or vital prognosis	2 months and 1 year
	Adverse events to amiodarone or beta-blockers		2 months and 1 year

*EKG* Electrocardiogram, *ICU* Intensive care unit, *LOS* Length of stay, *MAP* Mean arterial pressure

### Trial design {8}

The FAAC trial is a multicenter, prospective, randomized, controlled, single-blinded, two-arm study comparing landiolol to amiodarone for the treatment of atrial fibrillation following cardiac surgery. The FAAC trial was approved by the Ethics Committee of EST III with approval number 19.05.08 (registration number ID RDB: 2019-A00763-54). The FAAC trial is conducted in accordance with the Declaration of Helsinki, and the French laws [9]. The privacy of the participants and their personal medical records will be guaranteed by treating the data according to the French law n. 78–17 of 6 January 1978 and the European Union Data Protection Directive (95/46/EC24 October 1995).

A checklist of recommended items to address in a clinical trial protocol according to the Standard Protocol Items: Recommendations for Interventional Trials (SPIRIT 2013 Checklist) is provided in Additional file 1.

The approved initial version of FAAC is v2 14 05 2019 (ongoing version v5 28.09.2021); the recruitment began in January 2020. The estimated end of the study will be December 2024. Participants are currently being recruited and enrolled. The sponsor (CHU de Caen) is responsible for reporting any protocol modifications to the centers, to the ethic committees, and to the French Agency of Drug and Medication (ANSM).

## Methods: participants, interventions, and outcomes

### Study setting {9}

Patients are recruited in eleven French institutions (details in Additional file 2). The study sponsor is the Research and Innovation Department of the University Hospital of Caen, a public academic institution in France.

### Eligibility criteria {10}

Patients scheduled for cardiac surgery are eligible for the study.

Inclusion criteria are:

- Adult patients hospitalized in the cardiac intensive care unit after cardiac surgery including coronary artery bypass and/or aortic valve repair and/or ascending aorta surgery;
- New onset of postoperative atrial fibrillation persistent more than 30 min;
- Patient with a social security number;
- Patient with written informed consent.

Exclusion criteria are:

- Patients with hemodynamic instability with the need of an electrical cardioversion;
- Preoperative treatment with anticoagulant therapy;
- Contra-indication to amiodarone or beta-blockers;
- Sepsis;
- Slow PoAF (heart rate < 90/min);
- Patient with inotropic support;
- Patient history of atrial fibrillation;
- Emergency surgery, ventricular assist device, heart transplantation, TAVR, mechanical valve, mitral valve, or tricuspid valve repair.

### Who will take informed consent? {26a}

Patients are screened and informed during the consultation before surgery (conducted in France by the anesthesiologist, which is also the intensivist for the postoperative care), and they are screened and re-informed by the intensivist if POAF occur in the postoperative period and included after providing written consent. According to the French laws, only medical doctors recorded as investigators in the present study can inform and obtain written informed consent [9]. They should have validated an international council for harmonization for Good Clinical Practices and follow a specified formation for the study design of the FAAC study performed in initiation site meeting.

### Additional consent provisions for collection and use of participant data and biological specimens {26b}

Not applicable.

## Interventions

### Explanation for the choice of comparators {6b}

The FAAC trial will compare amiodarone and beta-blockers, which are the two most used therapeutics at bedside and largely recommended by learning societies [2, 3]. The landiolol was chosen as a beta-blocker because of its easy intravenous use, short delay of action, and rapid elimination.

### Intervention description {11a}

**Fig. 1:**
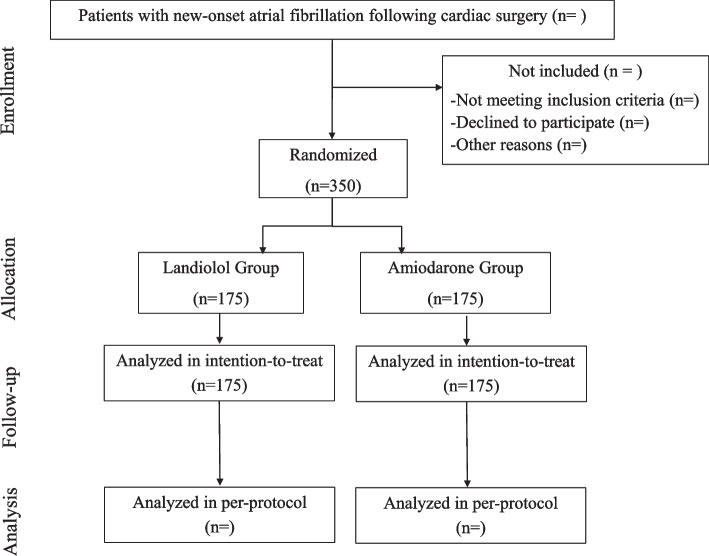
Flowchart of the FAAC study

Patients who underwent cardiac surgery and have a de novo persistent POAF (for at least 30 min) in the cardiac intensive care unit (ICU) and are eligible for the study are randomized after informed consent, after exclusion of hypovolemia and pericardial effusion with a transthoracic echocardiography and after exclusion of dyskalemia or anemia with an arterial blood gas performed in routine care. Randomization is performed by the anesthesiologist in charge of the patient in the cardiac ICU using CSOnline website (Ennov, Paris, France), 24/7 available. The randomization sequence is generated by the statistician of the study using permutated blocks and stratified by center. Patients are randomized in either the landiolol group or the amiodarone group (Fig. 1).

**Fig. 2:**
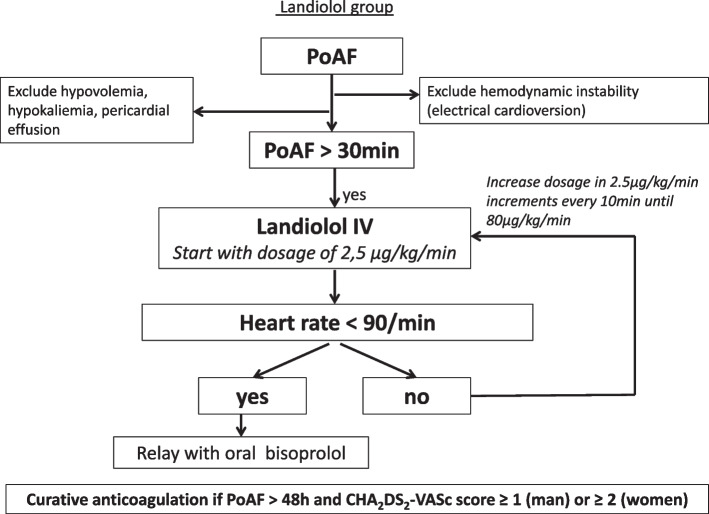
Landiolol group algorithm. IV, intravenous; PoAF, postoperative atrial fibrillation

In the landiolol group (Fig. 2), landiolol is administered intravenously without bolus at a starting dose of 2.5 μg/kg/min. This dose is increased by steps of 2.5 μg/kg/min every 10 min until a maximum dose of 80 μg/kg/min in order to reach a target heart rate (HR) of less than 90 beats per minute (bpm) [6]. Once this target heart rate is achieved, beta-blocker therapy is bridged within 24 h of landiolol infusion with bisoprolol 1.25 mg twice daily if the maximum dose of landiolol was inferior to 15 μg/kg/min or bisoprolol 2.5 mg twice daily if the maximum dose of landiolol was superior to 15 μg/kg/min (Additional file 3). Landiolol infusion is then discontinued.

**Fig. 3:**
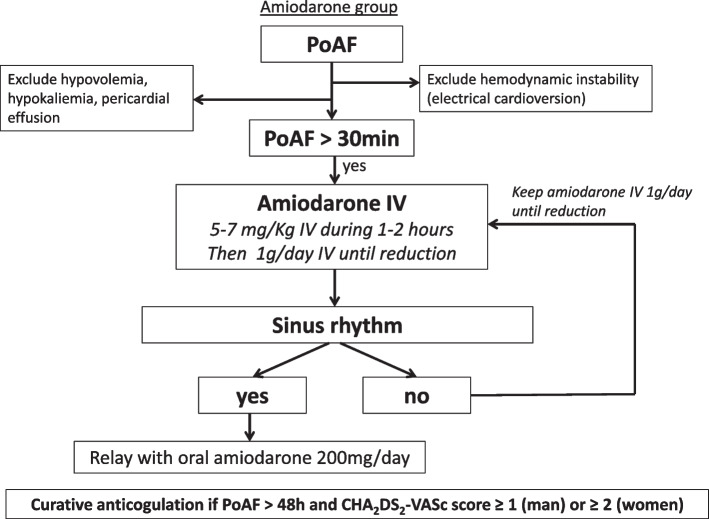
Amiodarone group algorithm. IV, intravenous; PoAF, postoperative atrial fibrillation

In the amiodarone group (Fig. 3), amiodarone is administered intravenously with a bolus of 5–7 mg/kg over 1 h and a subsequent continuous infusion of 1.0 g per day until reduction to sinus rhythm [3]. If reduction to sinus rhythm occurs or if the target heart rate drops below 90 beats per minute, intravenous infusion of amiodarone is discontinued and bridged via oral route with 200 mg amiodarone daily.

For both groups, the curative anticoagulation is started if PoAF is > 48 h and the CHA_2_DS_2_-VASc score is ≥ 1 (for men) or ≥ 2 (for women) [10]; the treatment (bisoprolol or amiodarone) is continued for 2 months, until cardiology consultation for evaluating the treatment and the anti-coagulation prescription [11].

### Criteria for discontinuing or modifying allocated interventions {11b}

Criteria for discontinuing the allocated treatment are extreme bradycardia (defined as heart rate < 40/min), arterial hypotension with MAP < 60 mmHg, or allergy with graduate 3 or 4 conducting to epinephrine use.

### Strategies to improve adherence to interventions {11c}

Patients are hospitalized, and therapeutics will be administered intravenously by nurses explaining that the attempt adherence will be excellent.

### Relevant concomitant care permitted or prohibited during the trial {11d}

All concomitant care and interventions are permitted during the trial. All patients have a continuous EKG, SpO_2_, and blood pressure monitoring during the intravenous treatment (amiodarone or landiolol). Usual care for postoperative cardiac surgery will be continued.

### Provisions for post-trial care {30}

No provisions are provided, because no ancillary and post-trial care is designed. Usual care will be conducted in the post-trial period.

The sponsor has insurance in accordance with the legal requirements in France. This insurance provides coverage for damage to research subjects through injury or death caused by the study. The insurance applies to the damage that becomes apparent during the study or within 10 years after the end of the study.

### Outcomes {12}

#### Primary outcome

The primary outcome is the number of patients (in percentage, 95% CI) in sinus rhythm 48 h after the first episode of PoAF after cardiac surgery. This primary outcome was chosen to compare the efficacy of both landiolol and amiodarone to reduce PoAF in the first 48 h which is the delay recommended to start the anticoagulation if PoAF is not reduced [3].

#### Secondary outcomes

The secondary outcomes are hemodynamic stability (hypotension, bradycardia), duration of stay in the ICU and in the hospital, number of patients (in percentage, 95% CI) with a recurrence of PoAF within 2 months and 1 year after surgery, number of patients (in percentage, 95% CI) with a thromboembolic complication within 2 months and 1 year after surgery, number of patients (in percentage, 95% CI) with a hemorrhagic complication within 2 months and 1 year after surgery, and number of patients (in percentage, 95% CI) with an adverse event due to landiolol or amiodarone within 2 months and 1 year after surgery.

### Participant timeline {13}

Additional file 4 summarizes the schematic diagram of study procedures.

### Sample size {14}

Two groups of 159 patients (318 patients in total) are needed to show an increase from 70 to 85% of patients in sinus rhythm 48 h after the first episode of PoAF [4, 8], using a two-sided α-risk at 5% and a power at 90%. Considering the uncertainty in the effect size, we need to include a total of 350 patients (175 patients per arm).

### Recruitment {15}

Recruitment will be conducted in collaboration with eleven public and private hospital in France, experienced with both amiodarone and landiolol use in clinical routine. The principal investigator and scientific responsible will introduce the study to healthcare staff (anesthesiologists, intensivists, cardiac surgeons, cardiologists, and nurses) with clinical research support, as part of their clinical team meetings in all recruiting organizations, and will tailor brochures targeted specifically for healthcare providers and patients. A newsletter will be realized and sent to all participants and healthcare providers in each participating hospital.

## Assignment of interventions: allocation

### Sequence generation {16a}

The biostatistician of the sponsor CHU de Caen will perform the randomization list. The randomization sequence is generated using permutated blocks of varying size and stratified by center, using R software version 4.0.5 (2021 The R Foundation for Statistical Computing). The randomization was centralized in a computer using the e-clinical platform.

### Concealment mechanism {16b}

There is no blinding in this open-label study (care providers know the group allocation, but the patient and the surgeon are not aware of the group allocation).

### Implementation {16c}

Randomization is performed by the anesthesiologist in charge of the patient in the cardiac ICU using CSOnline website (Ennov, Paris, France), 24/7 available.

## Assignment of interventions: blinding

### Who will be blinded {17a}

Patients are blinded of the study group. Care providers are aware of the group allocation.

### Procedure for unblinding if needed {17b}

As only patients were blinded of the allocation group, this is a simple blinded study.

## Data collection and management

### Plans for assessment and collection of outcomes {18a}

Data, patients’ consent, and outcomes will be recorded by the care provider in charge of the patient and a dedicated local research coordinator before and after enrollment using eCRFs (Ennov Clinical®).

### Plans to promote participant retention and complete follow-up {18b}

Data from patients who will be pulled out of the study because of refusal to participate, refusal of the follow-up evaluation, or because of any other reasons as decided by the main investigators will be analyzed according to their initial assigned group on an intention-to-treat analysis.

### Data management {19}

The data manager of the University Hospital of Caen is responsible for the development, sponsor database development, and data management.

Data will be collected and registered using electronic CRFs (Ennov Clinical®) in each center by dedicated local technical research. A research coordinator will centralize data from all sites.

### Confidentiality {27}

Study data will be collected only by authorized staff (study investigator, local research coordinator, or any person who has authorization as scheduled by the study protocol). Data will be stored in a local database accessible only to those who have authorization as scheduled by the study protocol. Access to the database will be with a personal login and password. Login to the database will be saved in the database’s login history folder.

The University Hospital of Caen conforms to the National Informatic and Liberty French Laws, and, therefore, patient confidentiality will be protected.

### Plans for collection, laboratory evaluation, and storage of biological specimens for genetic or molecular analysis in this trial/future use {33}

Not applicable.

## Statistical methods

### Statistical methods for primary and secondary outcomes {20a}

Categorical variables will be described as count, percentages, and 95% confidence interval, and continuous variables will be described as mean (standard deviation) or median [interquartile range], as appropriate. The analysis for the primary outcome will follow the intention-to-treat principle in which all the randomized patients will be analyzed in the assigned group, using the chi-square test or the Fisher exact test. Secondary outcomes will be analyzed with a chi-square test or a Fisher exact test for categorical variables and with a Student’s *t*-test or a Mann–Whitney *U* test for continuous variables according to their distributions. No sub-group analysis is planned. A two-tailed *p* < 0.05 is considered statistically significant. All statistical analysis will be conducted with SAS V9.4 (SAS Institute, Cary, NC).

### Interim analyses {21b}

No interim analysis planned.

### Methods for additional analyses (e.g., subgroup analyses) {20b}

There is no sub-group analysis planned. We are planning several sensitivity analyses including multiple imputations to deal missing data and potential lost to follow-up.

### Methods in analysis to handle protocol non-adherence and any statistical methods to handle missing data {20c}

Data from patients who will be pulled out of the study because of refusal to participate, refusal of the follow-up evaluation, or because of any other reasons as decided by the main investigators will be analyzed according to their initial assigned group on an intention-to-treat analysis. We are planning several sensitivity analyses including multiple imputations to deal missing data and potential lost to follow-up.

### Plans to give access to the full protocol, participant-level data, and statistical code {31c}

The final dataset will be available from the corresponding author upon request.

## Oversight and monitoring

No committee is planned for this trial.

### Composition of the coordinating center and trial steering committee {5d}

The coordinating center is the University Hospital of Caen, which is the sponsor of the FAAC study, including the main investigator (EC), scientific responsible (MOF), clinical research staff (CT), and biostatistics (AB, JJP). Their roles are firstly to ensure the patient’s right protection and data validity via quality control of the trial, in addition to management, analysis and interpretation of data, assistance in writing of the report, and support in the publication. Data collected are sponsor property, and every project of the publication has to be validated by the sponsor before submission and should include the sponsor in the list of authors.

No trial steering committee is formed.

### Composition of the data monitoring committee, its role and reporting structure {21a}

There is no safety monitoring board.

### Adverse event reporting and harms {22}

In France, the competent authority qualified this research as “research with minimal constraint.” This means that adverse events are not reported to the sponsor, only new safety information, i.e., if the investigator considers that the risk–benefit balance of the study should be reviewed.

### Frequency and plans for auditing trial conduct {23}

Not applicable.

### Plans for communicating important protocol amendments to relevant parties (e.g., trial participants, ethical committees) {25}

The sponsor University Hospital of Caen is responsible for reporting any protocol modifications to the centers, after the ethic committee’s approvals and ANSM authorization.

### Dissemination plans {31a}

We further plan to communicate these results to the anesthesiology review and to the congress.

## Discussion

PoAF frequently occurred after thoracic surgery, with an incidence between 20 and 50% of patients following cardiac surgery as reported in last recent studies [1]. Its physiopathology is multifactorial, but the hyperadrenergic response during the perioperative period (surgery-induced stress, hypovolemia, pain, anemia, hypoxemia, catecholamine administration) seems leading [1–3, 12]. This last point could explain that beta-blockers could be more adapted for PoAF than anti-arrhythmic drug as amiodarone. The best beta-blockers should have a short pharmacological effect to be quickly adapted in the perioperative setting and an excellent hemodynamic tolerance. Landiolol seems interesting, because its pharmacology seems to have a fast onset (1 min) and short half-life (4 min). Due to its S-enantiomer conformation, landiolol has less of a negative inotropic effect than esmolol (only other available intravenous beta-blocker) [6]. To date, only one retrospective study was conducted in one center with encouraging results in Japan for landiolol [8], justifying the present multicentric randomized study. That is why the main objective of this trial is to determine if landiolol could reduce more frequently the first episode of PoAF following cardiac surgery than amiodarone in the first 48 h. This delay of 48 h was chosen because it is clinically relevant in routine care to start anticoagulation to prevent stroke, according to the guidelines [11] and to the CHA_2_DS_2_-VASc score [10].

Some comments could be addressed concerning the limitations of the study. Because of our chosen endpoints, we had to exclude patients with preoperative atrial fibrillation and patients who are already under anticoagulant therapy or will require anticoagulant therapy because of implantation of a mechanical prosthetic valve or ventricular assist device. We also excluded patients scheduled to have a mitral valve or tricuspid valve repair, because in these patients, PoAF is not only the consequence of a hyperadrenergic state but also a cardiac anatomical and physiological modification with frequent left atrium enlargement [13] and a high incidence of PoAF [14]. Finally, we excluded patients with hypotension and patients with inotropic support, because beta-blocker therapy is contra-indicated in these instances. Our study population is limited to patients with PoAF after coronary artery bypass, no mechanical aortic valve repair and ascending aortic surgery with no history of atrial fibrillation, and no preoperative or scheduled postoperative treatment with anticoagulant therapy. Thus, the results of our study might not be applicable in these other instances.

## Trial status

Patients from 11 French cardiac surgery centers could be included. The recruitment began in January 2020, and the estimated end of the study will be on December 2024. Participants are currently being recruited and enrolled. Recruitment has not been completed at the time of this submission.

Protocol approval from the ethical committee, financial support, and eCRF were developed in 2019. Inclusions of patients were planned from 2020 to 2024. The database could be closed at the end of 2024 and be followed by data analysis, manuscript writing, submission for publication, and final report redaction as required by the authority.

## Supplementary Information

**Additional file 1. **SPIRIT Checklist.

**Additional file 2. **Listing of institutions.

**Additional file 3. **Protocol use of landiolol and bisoprolol.

**Additional file 4. **Schematic diagram of study procedure.

## Data Availability

The datasets analyzed during the current study and the statistical code are available from the corresponding author on reasonable request, as well as the full protocol.

## Abbreviations

PoAF: Postoperative atrial fibrillation
ICU: Intensive care unit
Bpm: Beats per minute
HR: Heart rate

## References

[1] Hindricks G, Potpara T, Dagres N, Arbelo E, Bax JJ, Blomström-Lundqvist C (2020). 2020 ESC Guidelines for the diagnosis and management of atrial fibrillation developed in collaboration with the european association for cardio-thoracic surgery (EACTS). Eur Heart J.

[2] American College of Cardiology Foundation; American Heart Association; European Society of Cardiology; Heart Rhythm Society, Wann LS, Curtis AB, Ellenbogen KA, Estes NA, Ezekowitz MD, Jackman WM, et al. Management of patients with atrial fibrillation (compilation of 2006 ACCF/AHA/ESC and 2011 ACCF/AHA/HRS recommendations): a report of the American College of Cardiology/American Heart Association Task Force on practice guidelines. Circulation. 2013;127:1916–26. 10.1161/CIR.0b013e318290826d 23545139

[3] Kirchhof P, Benussi S, Kotecha D, Ahlsson A, Atar D, Casadei B, et al. ESC Scientific Document Group. 2016 ESC Guidelines for the management of atrial fibrillation developed in collaboration with EACTS. Eur Heart J. 2016;37:2893–2962. 10.1093/eurheartj/ehw210 27567408

[4] Gillinov AM, Bagiella E, Moskowitz AJ, Raiten JM, Groh MA, Bowdish ME (2016). Rate control versus rhythm control for atrial fibrillation after cardiac surgery. N Engl J Med.

[5] Fischer MO, Fellahi JL, Lorne E (2016). Rate control or rhythm control for atrial fibrillation after heart surgery. N Engl J Med.

[6] Fellahi JL, Heringlake M, Knotzer J, Fornier W, Cazenave L, Guarracino F (2018). Landiolol for managing atrial fibrillation in post-cardiac surgery. Eur Heart J Suppl.

[7] Sezai A, Shiono M (2014). The role of β-blockers in cardiac perioperative management. Ann Thorac Cardiovasc Surg.

[8] Shibata SC, Uchiyama A, Ohta N, Fujino Y (2016). Efficacy and safety of landiolol compared to amiodarone for the management of postoperative atrial fibrillation in intensive care patients. J Cardiothorac Vasc Anesth.

[9] Toulouse E, Lafont B, Granier S, Mcgurk G, Bazin JE (2020). French legal approach to patient consent in clinical research. Anesthe Crit Care Pain Med.

[10] Lip GY, Nieuwlaat R, Pisters R, Lane DA, Crijns HJG (2010). Refining clinical risk stratification for predicting stroke and thromboembolism in atrial fibrillation using a novel risk factor-based approach: the euro heart survey on atrial fibrillation. Chest.

[11] January CT, Wann LS, Calkins H, Chen LY, Cigarroa JE, Cleveland JC (2019). 2019 AHA/ACC/HRS Focused Update of the 2014 AHA/ACC/HRS Guideline for the Management of Patients With Atrial Fibrillation: A Report of the American College of Cardiology/American Heart Association Task Force on Clinical Practice Guidelines and the Heart Rhythm Society. J Am Coll Cardiol.

[12] Maesen B, Nijs J, Maessen J, Allessie M, Schotten U (2012). Post-operative atrial fibrillation: a maze of mechanisms. Europace.

[13] Kawczynski MJ, Gilbers M, Van De Walle S, Schalla S, Crijns HJ, Maessen JG (2021). Role of pre-operative transthoracic echocardiography in predicting post-operative atrial fibrillation after cardiac surgery: a systematic review of the literature and meta-analysis. Europace.

[14] O'Brien B, Burrage PS, Ngai JY, Prutkin JM, Huang CC, Xu X (2019). Society of cardiovascular anesthesiologists/European association of cardiothoracic anaesthetists practice advisory for the management of perioperative atrial fibrillation in patients undergoing cardiac surgery. Anesth Analg.

