# Supplementary material for: Comparison of landiolol and amiodarone for the treatment of new-onset atrial fibrillation after cardiac surgery (FAAC) trial: study protocol for a randomized controlled trial
Source: Trials. 2023 May 25;24:353. doi: 10.1186/s13063-023-07353-6 (PMC10210392; doi:10.1186/s13063-023-07353-6)
Supplement: Supplementary file 2 — Additional file 2. Listing of institutions. [file 13063_2023_7353_MOESM2_ESM.docx]

Additional file 2 Listing of institutions

| Number | Center | Location | Country |
| --- | --- | --- | --- |
| 1 | University Hospital of Caen | Caen | France |
| 2  3  4  5  6  7  8  9  10  11 | University Hospital of Dijon  University Hospital of Nantes  University Hospital of Brest  Hôpital universitaire Louis Pradel  Clinique Saint Augustin  Clinique du Millénaire  University Hospital of Amiens  University Hospital of Rouen  University Hospital of Lille  Hôpital Henri Mondor | Dijon  Nantes  Brest  Lyon  Bordeaux  Montpellier  Amiens  Rouen  Lille  Créteil | France  France  France  France  France  France  France  France  France  France |
