# Supplementary material for: Comparison of landiolol and amiodarone for the treatment of new-onset atrial fibrillation after cardiac surgery (FAAC) trial: study protocol for a randomized controlled trial
Source: Trials. 2023 May 25;24:353. doi: 10.1186/s13063-023-07353-6 (PMC10210392; doi:10.1186/s13063-023-07353-6)
Supplement: Supplementary file 3 — Additional file 3. Protocol use of landiolol and bisoprolol. [file 13063_2023_7353_MOESM3_ESM.docx]

**Landiolol and bisoprolol uses**

Landiolol seringue : Reconstitute a 300mg vial in a 50mL syringe with NaCl 0.9% = 6mg/mL

| Weight of patient | 2,5µg/kg/min with 6mg/ml |  |
| --- | --- | --- |
| 40 | 1 | ml/h |
| 50 | 1.25 | ml/h |
| 60 | 1.5 | ml/h |
| 70 | 1.75 | ml/h |
| 80 | 2 | ml/h |
| 90 | 2.25 | ml/h |
| 100 | 2.5 | ml/h |

**PoFA**

**Exclude hypovolemia, hypokaliemia, pericardial effusion**

Exclude hemodynamic instability (electrical cardioversion)

**STOP Landiolol if:**

HR < 40/min

MAP < 60 mmHg

Bronchospasm

*Increase dosage in 2.5μg/kg/min increments every 10min until 80μg/kg/min*

**NO**

**YES**

**Heart rate < 90/min**

**PoFA > 30min**

**Curative anticoagulation if PoAF > 48h and CHA_2_DS_2_-VASc score ≥ 1 (man) or ≥ 2 (women)**

**Landiolol IV**

*Start with dosage of 2,5 μg/kg/min*

**Start oral bisoprolol in relay and wait 2 hours before ↘ landiolol**

**↘ landiolol** by decrements of 2,5µg/kg/min every 10 min

**Bisoprolol** 1,25mg x 2/day if landiolol ≤ 15 µg/kg/min (or 2,5mg x 2/day if > 15 µg/kg/min)

And again if failure of ↘ landiolol (maximal posology of 10mg/day)
