# Supplementary material for: Comparison of landiolol and amiodarone for the treatment of new-onset atrial fibrillation after cardiac surgery (FAAC) trial: study protocol for a randomized controlled trial
Source: Trials. 2023 May 25;24:353. doi: 10.1186/s13063-023-07353-6 (PMC10210392; doi:10.1186/s13063-023-07353-6)
Supplement: Supplementary file 4 — Additional file 4. Schematic diagram of study procedure. [file 13063_2023_7353_MOESM4_ESM.docx]

**Add file 4 schematic diagram of study procedures**

|  | Anesthesia consultation | PoAF | PoAF > 30min | Follow until hospital discharge | 2 months | 1 year |
| --- | --- | --- | --- | --- | --- | --- |
| Oral and written information | X |  |  |  |  |  |
| Eligibility criteria | X | X | X |  |  |  |
| Written consent |  | X |  |  |  |  |
| Randomization |  |  | X |  |  |  |
| Clinical care (MAP, heart rate, all vital signs) |  | X | X | X |  |  |
| Complications |  |  |  | X | X | X |
| Treatments tolerance |  |  |  | X | X | X |
| Redux of PoAF |  |  |  | X | X | X |
| Evaluation of rhythm |  |  |  | X | X | X |
| Thrombo-embolic events |  |  |  | X | X | X |
| Hemorrhagic complications |  |  |  | X | X | X |

MAP: mean arterial pressure; PoAF, postoperative atrial fibrillation.
